# Gene Targeting in the Red Alga *Cyanidioschyzon merolae*: Single- and Multi-Copy Insertion Using Authentic and Chimeric Selection Markers

**DOI:** 10.1371/journal.pone.0073608

**Published:** 2013-09-05

**Authors:** Takayuki Fujiwara, Mio Ohnuma, Masaki Yoshida, Tsuneyoshi Kuroiwa, Tatsuya Hirano

**Affiliations:** 1 Chromosome Dynamics Laboratory, RIKEN, Wako, Saitama, Japan; 2 Faculty of Science, Rikkyo University, Toshima-ku, Tokyo, Japan; 3 Faculty of Life & Environmental Sciences, University of Tsukuba, Tsukuba, Ibaraki, Japan; 4 Core Research for Evolutional Science and Technology (CREST), Japan Science and Technology Agency, Chiyoda-ku, Tokyo, Japan; Cancer Research UK London Research Institute, United Kingdom

## Abstract

The unicellular red alga *Cyanidioschyzon merolae* is an emerging model organism for studying organelle division and inheritance: the cell is composed of an extremely simple set of organelles (one nucleus, one mitochondrion and one chloroplast), and their genomes are completely sequenced. Although a fruitful set of cytological and biochemical methods have now been developed, gene targeting techniques remain to be fully established in this organism. Thus far, only a single selection marker, *URA_Cm-Gs_*, has been available that complements the uracil-auxotrophic mutant M4. *URA_Cm-Gs_*, a chimeric *URA5.3* gene of *C. merolae* and the related alga *Galdieria sulphuraria*, was originally designed to avoid gene conversion of the mutated *URA5.3* allele in the parental strain M4. Although an early example of targeted gene disruption by homologous recombination was reported using this marker, the genome structure of the resultant transformants had never been fully characterized. In the current study, we showed that the use of the chimeric *URA_Cm-Gs_* selection marker caused multicopy insertion at high frequencies, accompanied by undesired recombination events at the targeted loci. The copy number of the inserted fragments was variable among the transformants, resulting in high yet uneven levels of transgene expression. In striking contrast, when the authentic *URA5.3* gene (*URA_Cm-Cm_*) was used as a selection marker, efficient single-copy insertion was observed at the targeted locus. Thus, we have successfully established a highly reliable and reproducible method for gene targeting in *C. merolae.* Our method will be applicable to a number of genetic manipulations in this organism, including targeted gene disruption, replacement and tagging.

## Introduction

The red alga *Cyanidioschyzon merolae* is a small (2 µm in diameter), unicellular, photoautotrophic organism that inhabits sulphate-rich hot springs. The organization of a *C. merolae* cell is very simple: it contains one each of the nucleus, the mitochondrion and the chloroplast, but has no rigid cell wall. The cell also has a minimum set of membranous structures: simple architectures of the endoplasmic reticulum and the Golgi body, a single microbody (peroxisome), and a small number of lysosomes (vacuoles) [1]. The three major organelles divide in a sequential and coordinated manner (in the order of the chloroplast, the mitochondrion and the nucleus), and their division cycles can readily be synchronized by cultivation under light and dark cycles [2], having led to their extensive characterizations at the biochemical and cytological levels [3]. Equally important, the genomes of the three organelles have completely been sequenced, revealing that *C. merolae* has the simplest nuclear genome among photosynthetic eukaryotes analyzed so far [4]–[7]. These simple features make microarray and proteome analyses straightforward, and help analyze a number of cellular processes such as organelle divisions and cellular metabolisms [8]–[10]. The simple genome organization (e. g., a small number of introns and transposable elements, and simple centromere sequences without surrounding repetitive DNAs) also makes *C. merolae* an excellent model organism for studying chromosomal organization and genomic evolution.

**Figure 1:**
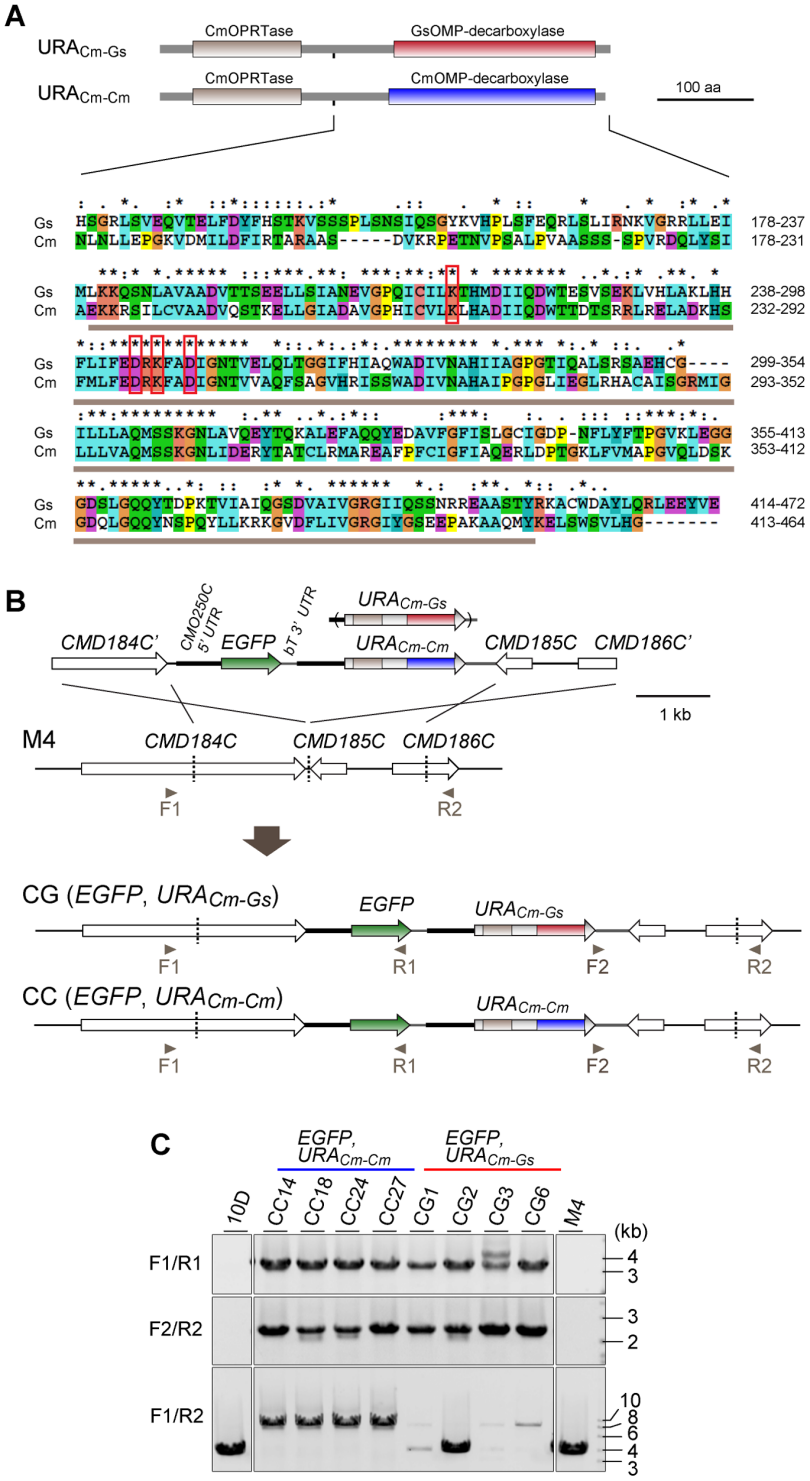
Targeted insertion of *EGFP* by homologous recombination using *URA_Cm-Cm_* and *URA_Cm-Gs_* as selection markers. (**A**) Schematic diagram of the domain architectures of the two selection markers is shown on the top. Alignment of amino-acid sequences surrounding the OMP-decarboxylase domain of *C. merolae* and *G. sulphuraria* is shown in the bottom. The gray bar indicates the conserved OMP-decarboxylase domain. The red squares indicate amino-acid residues that play key roles in the enzymatic activity of OMP-decarboxylase [20]. (**B**) Schematic diagrams of targeted gene insertion by homologous recombination. The first line indicates the introduced DNA fragment, whereas the second line indicates the genomic structure of the parental strain M4. For efficient expression of *EGFP*, the 5′-UTR of the *CMO250C* gene and the 3′-UTR of the *β-tubulin* gene were utilized as a promoter and a putative polyadenylation signal sequence, respectively. The third and fourth lines indicate the predicted genomic structures in which a single copy is inserted by double-crossover homologous recombination in each case. The arrowheads indicate the positions of PCR primers used. (**C**) PCR analysis of CC and CG strains isolated independently, along with 10D (wild-type strain) and M4 (parental strain), to confirm homologous recombination events. Primers used were F1 (No. 25), R1 (No. 26), F2 (No. 27) and R2 (No. 28) shown in Table S1. The predicted sizes of PCR products are as follows: F1/R1, 3.4 kb for CC and CG, no band for 10D and M4; F2/R2, 2.4 kb for CC and CG, no band for 10D and M4; F1/R2, 8.2 kb for CC and CG, 3.9 kb for 10D and M4.

Despite the great potential for contributing to many areas in cell biology, recombinant DNA technologies available in *C. merolae* remain very limited [11]–[13]. In this organism, currently available is a single selection marker, the *URA5.3* gene (*CMK046C*), which encodes a protein composed of the orotidine-5′-phosphoribosyltransferase (OPRTase) domain and the orotidine 5′-monophosphate (OMP)-decarboxylase domain [14] (Figure 1A). The uracil-auxotrophic mutant strain M4 has a point mutation in the second domain. An early study had noticed that, when the authentic *URA5.3* gene (hereafter referred to as *URA_Cm-Cm_*) was introduced on a plasmid into M4, the introduced wild-type gene repaired the mutated allele in the genome, presumably through gene conversion [14]. This observation promoted the subsequent study to use a chimeric *URA5.3* gene composed of *C. merolae* and *G. sulphuraria* sequences (hereafter referred to as *URA_Cm-Gs_*) as a selection marker for the purpose of gene targeting on the genome [15](Figure 1A). The authors reasoned that the *G. sulphuraria* OMP-decarboxylase domain retains the same level of enzymatic activity as that of its *C. merolae* counterpart, but is not homologous enough to support gene conversion of the mutated *URA5.3* allele in the M4 strain. This approach allowed the authors to demonstrate the first successful example of targeted gene disruption by homologous recombination in this organism, which was confirmed by PCRs [15]. It should be noted, however, that the genome structure was not analyzed by Southern blotting in this study, leaving the possibility open that unintended recombination events, such as multicopy insertion and/or additional insertion into untargeted loci, had occurred. In fact, more recent trials of our own gene targeting using *URA_Cm-Gs_* as a selection marker, followed by Southern blotting analyses of the resultant strains, provided evidence for insertion of variable copies of the transgene into the targeted locus. There had been no previous example of targeting gene disruption or insertion experiments using the authentic *URA5.3* gene (*URA_Cm-Cm_*) as a selection marker. For this reason, we decided to revisit this issue in the current study by directly comparing the ability of *URA_Cm-Cm_* and *URA_Cm-Gs_* as selection markers in *C. merolae*. Our results revealed hitherto-unrecognized functional differences between the two selection markers, and firmly established a highly reliable and reproducible method for gene targeting in *C. merolae*.

## Results and Discussion

### Experimental Designs

To quantitatively validate the ability of *URA_Cm-Cm_* and *URA_Cm-Gs_* as selection markers for targeted gene insertion, we constructed a pair of gene cassettes, each containing the *EGFP* transgene and one of the selection markers, which was designed to be targeted into the convergent intergenic region of *CMD184C* and *CMD185C* (Figure 1B). This locus was selected as the target site because it was very short and was not expected to contain promoter activities that potentially affect gene expression in its vicinity. Shown in Figure 1B is the basic strategy used for one-step insertion of the *EGFP* gene and selection markers by homologous recombination. Colony PCRs using the primer set F1/R1 were set up as a primary screen for strains in which the left arm of the introduced fragment had been recombined at the *CMD184C* locus on the genome. We found that 35% of the colonies screened (38/108) produced a PCR fragment with an expected length in a transformation experiment using *URA_Cm-Cm_* as a selection marker. In contrast, only 10% (11/108) were positive when *URA_Cm-Gs_* was used instead. Among these positive clones, we selected four independent, uracil-prototrophic strains from each marker. The resultant transformants were named CC strains for *URA_Cm-Cm_* (CC14, CC18, CC24, and CC27), and CG strains for *URA_Cm-Gs_* (CG1, CG2, CG3, and CG6). We used specific pairs of primers for PCRs and verified the occurrence of the recombination events at the *CMD184C* region (Figure 1C, F1/R1) and the *CMD185C*/*CMD186C* region (Figure 1C, F2/R2) in both CC and CG strains. However, when PCRs were performed using another pair of primers that was designed to amplify the whole inserted regions, different products were amplified between CC and CG strains (Figure 1C, F1/R2). In all CC strains, a single-sized fragment with an expected length of 8.2 kb was amplified, demonstrating that single-copy insertion events had occurred. In contrast, the expected 8.2-kb fragment was poorly or rarely amplified from CG strains. Moreover, the strains CG1 and CG2 additionally produced a fragment of 3.9 kb, the same length amplified from the wild-type (10 D) and parental (M4) strains. These results suggested that single-copy insertion rarely occurred with the construct containing the chimeric selection marker, yielding a mixture of revertants and undesired transformants.

### The Authentic Selection Marker URA_Cm-Cm_ Guarantees Single-copy Insertion whereas the Chimeric Marker URA_Cm-Gs_ Causes Multicopy Insertion

**Figure 2:**
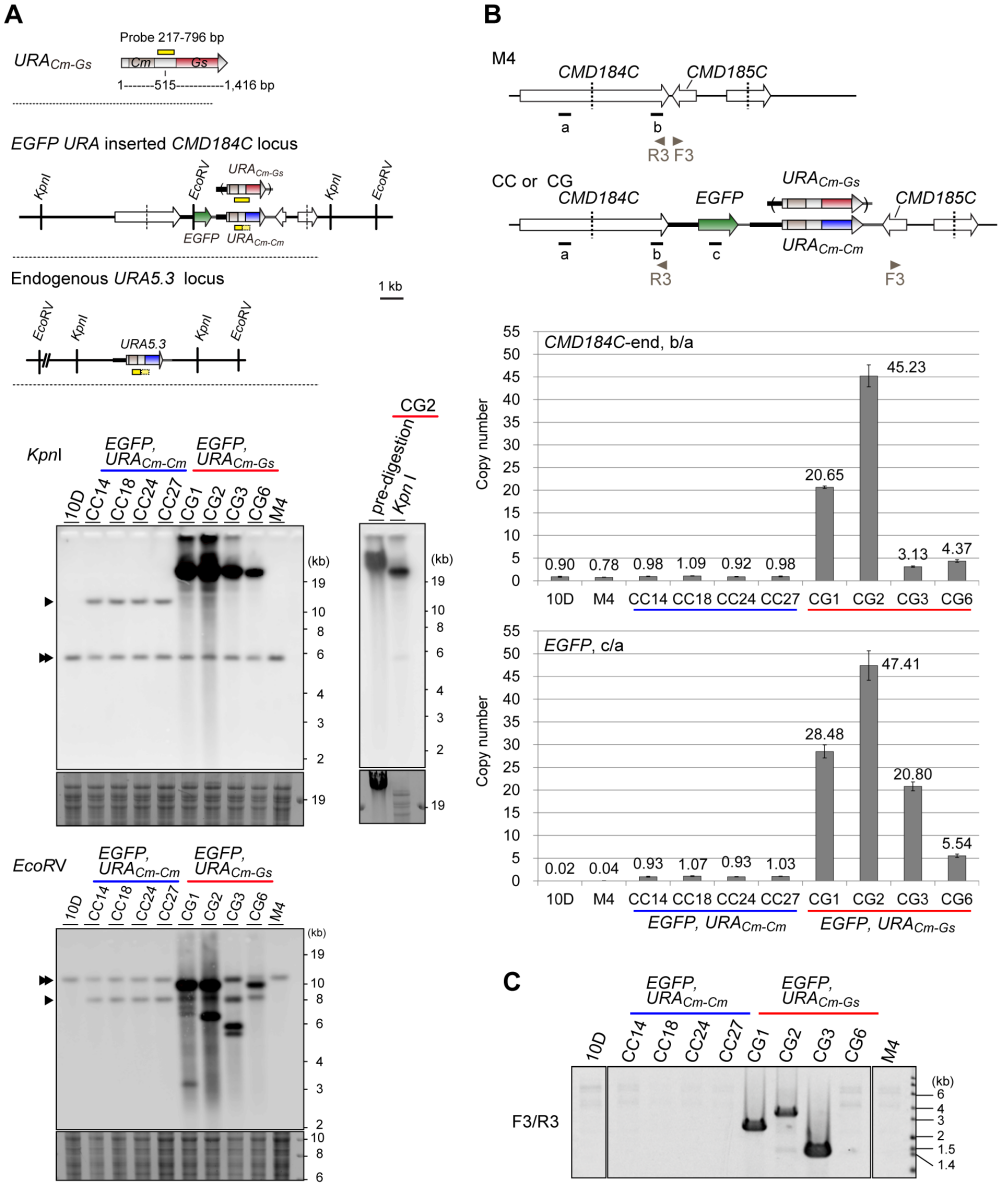
Characterization of the genome structure of CC and CG strains. (**A**) Southern blotting analyses of CC strains (CC14, CC18, CC24 and CC27) and CG strains (CG1, CG2, CG3 and CG6) along with 10 D and M4. Schematic representations include the position of the probe (yellow bars) and restriction enzyme sites used in the Southern blotting analyses. The *URA_Cm-Gs_* gene is a chimera of *C. merolae* (1 to 515 bp) and *G. sulphuraria* (516 to 1416 bp) sequences. The hybridization probe recognizes the introduced fragments (both *URA_Cm-Gs_* and *URA_Cm-Cm_*) as well as the endogenous *URA5.3* gene. The arrowhead indicates the predicted position of a fragment produced by a single-copy-insertion event. The double arrowhead indicates the predicted position of a fragment derived from the endogenous *URA5.3* locus. The predicted sizes of these fragments are as follows: *Kpn*I, 12.4 kb for CC and CG, 5.8 kb for endogenous *URA5.3*; *Eco*RV, 8.2 kb for CC and CG, 11.4 kb for endogenous *URA5.3*. A part of the ethidium bromide (EtBr)-stained gel is shown as a loading control. (**B**) Quantitative-PCR analyses of CC and CG strains, along with 10 D and M4, to estimate the copy number of *CMD184C*-end (b) and *EGFP* (c). The value of (b) and (c) in each strain was normalized against that of *CMD184C*-mid (a), located outside of the introduced DNA fragment, to estimate their copy number. The bars indicate the SD (n = 3). (**C**) PCR analysis to detect tandem insertion. The positions of PCR primers (F3 and R3) are shown in (**B**) and their sequences were shown in Table S1 with No. 29 and No. 30, respectively.

We then analyzed the genomic structures of CC and CG strains by Southern blotting analyses. A hybridization probe was designed to recognize both *URA_Cm-Cm_* and *URA_Cm-Gs_* as well as the endogenous *URA5.3* gene. The first set of analyses against *Kpn*I-digested genomic fragments identified a 5.8-kb band corresponding to the endogenous *URA5.3* locus in all CC and CG strains (Figure 2A, *Kpn*I, double arrowhead). This result demonstrated that neither construct altered the genomic structures of the endogenous *URA5.3* locus. In CC strains, an additional ∼12-kb fragment with the same intensity was detected (Figure 2A, *Kpn*I, single arrowhead), indicating that the predicted single-copy insertion via a double-crossover reaction had occurred at the *CMD184C* locus. In contrast, larger fragments were observed in CG strains, implicating multicopy insertion of the introduced fragments. Moreover, the copy number was apparently variable among these CG strains as judged by their signal intensities. Southern blotting against undigested and *Kpn*I-digested genomic DNA confirmed that the large-sized fragment observed in CG2 was not an artifact derived from incomplete digestion of genomic DNA (Figure 2A, CG2). The second set of analyses against *Eco*RV-digested genomic fragments further confirmed predicted single-copy insertion in CC strains, and multicopy insertion in CG strains. The size of the positive fragments was variable among different CG strains.

To rigorously estimate the inserted copy number in each strain, we performed quantitative-PCR analyses. The copy number of the *CMD184C*-end sequence (Figure 2B, b) and the *EGFP* sequence (Figure 2B, c) in each strain was estimated by being normalized against the *CMD184C*-mid sequence (Figure 2B, a) that is located outside of the introduced DNA fragment. We found that CC strains contained ∼1 copy each of the *CMD184C*-end and *EGFP* sequences (Figure 2B, graphs), a result perfectly consistent with that obtained by Southern blotting (Figure 2A). In striking contrast, the estimated copy numbers of *CMD184C*-end and *EGFP* in the CG strains were high and variable among the different strains (from ∼3 up to ∼48 copies). Moreover, the estimated numbers of the *CMD184C*-end and *EGFP* sequences sometimes diverge (e. g., in CG3). We then designed and performed a PCR analysis to seek for the possibility of tandemly repeated insertion of the introduced fragments (Figure 2C). If the fragments were inserted into a tandem manner, this analysis would detect amplification of a ∼3-kb fragment. As expected, no such bands were amplified from the control and CC strains. In the CG strains, however, positive bands with different lengths and different strengths were amplified, suggesting that radically rearranged sets of the fragments were inserted into the genome in these strains. The current data do not eliminate the possibility that some of the introduced fragments might be inserted into loci outside of the targeted region (i.e., *CMD184C*/*CMD185C*).

### The Copy Number of Gene Insertion Closely Reflects Transgene Expression

**Figure 3:**
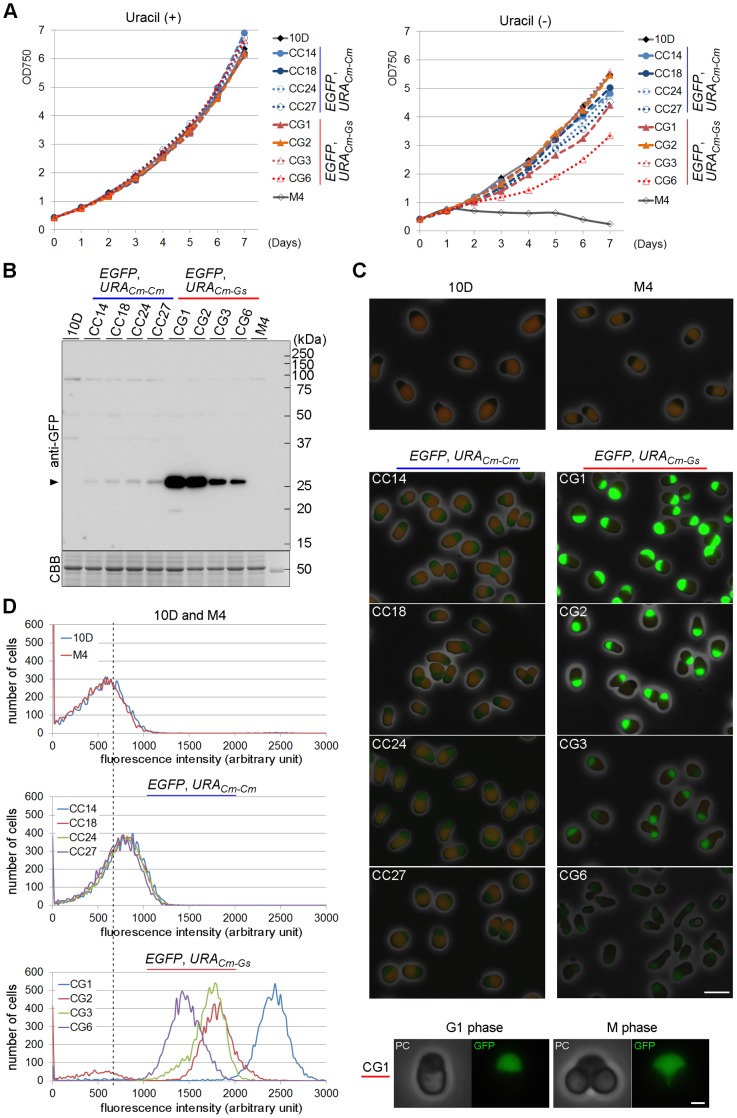
Growth of and EGFP expression from the transformants. (**A**) Growth curves of the CC and CG strains in MA2 medium in the presence or absence of uracil. (**B**) Immunoblotting analysis against total lysates with anti-GFP. The arrowhead indicates the position of EGFP. A CBB-stained part of the gel is shown as a loading control. (**C**) Fluorescent images showing EGFP signals detected in the CC and CG strains. The CG strains emitted much higher levels of EGFP signals than the CC strains. Exposure times under blue-light excitation are 0.5 sec for 10 D, M4 and CC stains, and 0.2 sec for CG strains. The lower panels show typical G1 and M phase cells (CG1), indicating that the EGFP signals distributed throughout the cytosol. Note that the chloroplasts emitted red autofluorescence. Bars, 5 µm (upper panels) and 1 µm (lower panels). (**D**) Flow cytometry analysis of EGFP fluorescence. The broken line indicates the mode value calculated from the 10 D data, representing the sum of background and autofluorescence signals.

Having examined the genomic property of the transformants, we next investigated the growth property of each strain. It was found that all transformants displayed growth curves indistinguishable from those of M4 (parental strain) and 10 D (wild type) in MA2 medium containing uracil (Figure 3A, left). In MA2 medium containing no uracil, the uracil-auxotrophic strain M4 failed to grow, but all four CC strains grew almost normally as the wild-type strain (Figure 3A, right). The growth rates of CG strains were variable, with a tendency that strains with low-copy-number insertion grew slowly (e. g., CG6).

We then looked at the expression level of the EGFP transgene in each strain. Immunoblotting analysis demonstrated that a constant level of EGFP expression was detectable in CC strains, whereas much higher levels of expression were observed in CG strains (Figure 3B). The expression levels in CG strains were highly variable, yet largely proportional to the estimated copy numbers inserted into the genome. Consistently, fluorescence microscopy showed a low and constant level of signals was observed among CC strains, whereas higher and variable levels were detectable in CG strains (Figure 3C). The EGFP signals distributed throughout the cytoplasm in both G1 phase and M phase (Figure 3C, bottom). Finally, flow cytometry analysis of the EGFP signals also substantiated the immunoblotting data (Figure 3D). The fluorescent intensities in CG strains were largely correlated with the gene copy numbers estimated, with an exception of CG2. Although the exact reason for this discrepancy is unknown, it is possible, for instance, that scrambled organization of multiple copies of the transgene on the genome affected the level of protein expression from each copy.

### Conclusions and Perspectives

In the current study, we have firmly established a technique of targeted gene insertion in *C. merolae*, demonstrating that the use of the authentic *URA_Cm-Cm_* selection marker guarantees single-copy insertion into the genome. It is important to emphasize that modified versions of this technique have now been successfully applied to targeted disruption and targeted replacement (tagging) of condensin genes [16]. On the other hand, the chimeric *URA_Cm-Gs_* gene, a selection marker used in the previous studies, is found to result in multicopy insertion at high frequencies. We reason that the activity of *G. sulphuraria* OMP-decarboxylase is substantially lower than that of the *C. merolae* counterpart in *C. merolae* cells, and that multicopy insertion of *URA_Cm-Gs_* is positively selected for rescuing the mutant allele of M4. It is anticipated that the current results will substantially simplify and strongly encourage future genetic analyses in *C. merolae*, thereby facilitating our understanding of the fundamental mechanisms of cell proliferation, organelle inheritance and adaptation to stressful environments. Moreover, the technique established here will open an avenue to regulated overexpression of transgenes and may help create cellular metabolites potentially useful for the development of next-generation biofuels that utilize algal ability for carbon fixation.

## Materials and Methods

### Cell Cultures

The wild-type *C. merolae* strain 10D-14 [17] and the uracil-auxotrophic mutant M4 [14] were used in the current study. They were maintained by gyratory culture (130 rpm), in either 2× Allen’s medium [18] or MA2 medium [11] containing uracil (0.5 mg/mL), at pH 2.3 and 42°C under continuous light. Cell cultures were diluted to an OD_750_ of 0.4, incubated under continuous light with aeration for 24 h, and then used for downstream analyses.

### Targeted Gene Insertion

Primers used for plasmid construction are listed in Table S1. To make the *URA_Cm-Cm_* selection marker, a DNA fragment containing the *URA5.3* gene (including an 897-bp upstream sequence and a 471-bp downstream sequence) was amplified by PCR using the primer set No. 1/2 and *C. merolae* 10 D genomic DNA as a template. A *CMD184C* fragment (containing the last 1,961 bp of the *CMC184C* ORF and its 1.9-kb downstream sequence) and a pQE80 vector were amplified by PCR with the primer sets No. 3/4 and No. 5/6, respectively. The *CMD184C* fragment was subcloned into pQE80 vector (QIAGEN) using the In-Fusion HD Cloning Kit (Clontech). The resultant pD184 vector was amplified by PCR with the primer set No. 7/8. An upstream sequence of *CMO250C* (−600 to −1), the *EGFP* ORF (Takara Bio Inc.), and a downstream sequence of *β-tubulin* (+1 to +200) were amplified by PCR with the primer sets No. 9/10, No. 11/12, and No. 13/14, respectively, and subcloned into pQE vector using the In-Fusion HD Cloning Kit. Using the resultant pO250-EGFP vector as the template DNA, the assembled fragment (the upstream region of CMO250C [−600 to −1], *EGFP* ORF, the downstream of *β-tubulin* [+1 to +200]) was amplified by PCR with the primer sets No. 15/16, and subcloned into amplified pD184 vector. The resultant pD184-O250-EGFP vector was amplified with the primer sets No. 17/18. The *URA_Cm-Cm_* selection marker was subcloned into the amplified pD184-O250-*EGFP* vector. The resultant pD184-O250-*EGFP*-*URA_Cm-Cm_* vector was used as a template to amplify the assembled set of fragments (containing a part of the *CMD184C* ORF and 3′-UTR [1270 to +25], *CMO250C* 5′-UTR, *EGFP*, *CMO250C* 5′-UTR, *EGFP, β-tubulin* 3′-UTR, the *URA_Cm-Cm_* marker, and the *CMD184C* downstream sequence [+28 to +1,448]) with the primer set No. 19/20. The amplified 8.1-kb DNA fragment was used for transformation.

To remove the sequence containing the OMP-decarboxylase domain of *URA_Cm-Cm_* from the pD184-O250-*EGFP*-*URA_Cm-Cm_* vector, the vector was amplified by PCR with the primer set No. 21 and 22. The sequence containing the OMP-decarboxylase domain of *G. sulphuraria URA5.3* was amplified by PCR using the primer set No. 23/24 and *URA_Cm-Gs_* selection marker as the template DNA [15], and then the sequence was subcloned into the amplified pD184-O250-*EGFP*-*URA_Cm-Cm_* vector. The resultant pD184-O250-*EGFP*-*URA_Cm-Gs_* was used as a template to amplify the assembled fragment (containing a part of the *CMD184C* ORF and 3′-UTR [1270 to +25], *CMO250C* 5′-UTR, *EGFP*, *CMO250C* 5′-UTR, *EGFP, β-tubulin* 3′-UTR, the *URA_Cm-Gs_* marker, and the *CMD184C* downstream sequence [+28 to +1,448]) by PCR with the primer set No. 19/20 and the extremely high-fidelity PCR polymerase PrimeSTAR Max (Takara). The amplified 8.1-kb DNA fragment was used for transformation.

The *C. merolae* strain M4 [14] was used for targeted gene insertion as a parental strain. Cells were grown in MA2 medium [11] containing uracil (0.5 mg/ml) and 5-fluoroorotic acid (FOA) (0.8 mg/ml) in a Erlenmeyer flask with shaking at 130 rpm under continuous white light (80 µmol/s⋅m^2^)at 40°C. MA2 medium was solidified with 0.5% (w/v) gellan gum (Wako, Japan). To adjust pH to ∼2.3, H_2_SO_4_ was added at a final concentration of 0.05% (v/v). Transformation was performed as described previously [11], [15]. A 4-µg aliquot of the PCR fragment was used for transformation, and cells were incubated under continuous white light at 40°C until colonies were formed. The colonies were then transferred to starch on solidified MA2 medium containing uracil (0.5 mg/ml).

### Southern Blotting Analyses

Cells (10 ml, OD_750_ = 1.0) were harvested, resuspended in 4 ml of nucleic acid extraction buffer (100 mM Tris-HCl, pH 7.5, 50 mM EDTA, and 1% SDS) containing 25 µg/ml proteinase K, and incubated at 37°C for 12 hr. The lysate was extracted with PCI (phenol: chloroform: isoamylalcohol = 25∶24∶1), and total nucleic acids were precipitated with isopropanol in the presence of 20 µg of glycogen. The pellet was dissolved in TE containing RNase A (QIAGEN) at a final concentration of 0.5 mg/ml and incubated at 37°C for 45 min. The genomic DNA was then purified by PCI extraction, followed by isopropanol precipitation. 2 µg of genomic DNA were digested with *Kpn*I or *Eco*RV, and then separated by 0.8% agarose gel electrophoresis. Transferring DNA from the agarose gel to a nitrocellulose membrane was performed by a conventional alkaline method using 20×SSC buffer. DIG-labeled DNA fragments were prepared by PCR using DIG Probe Synthesis Kit (Roche) with specific primers (Table S1) and used as hybridization probes. The DIG was detected with alkaline phosphatase (AP)-conjugated anti-DIG antibody (Roche) and CDP-Star (Roche), and the signals were visualized with the luminescent image analyzer LAS-3000 (FUJIFILM).

### Quantitative PCRs

Real-time PCRs were performed using a CFX96 Real-Time PCR Detection System and C1000 Thermal Cycler (Bio-Rad) in a 20- µl reaction mixture containing 1 µl template DNA, 0.5 nM primers (Table S1) and 10 µl SsoFast EvaGreen Super Mix (Bio-Rad). Standard curves were constructed using serially diluted solutions of DNA isolated from *C. merolae* cells and the relevant sets of primers. The copy number of the (b) or (c) region in each strain was normalized with the data of the (a) region that is located outside of the introduced DNA fragment (Figure 2B).

### Growth Curves

To draw growth curves of *C. merolae* strains, they were diluted to an OD_750_ of 0.4 in MA2 medium containing uracil, and were grown for a week in order to equalize their growth conditions. The cells were then inoculated in the medium at an OD_750_ of 0.2, and their growth was periodically monitored. Alternatively, the cells were washed twice with a uracil-free MA2 medium, and then inoculated in the uracil-free medium at an OD_750_ of 0.2, and their growth was periodically monitored.

### Immunoblotting Analysis

A whole cell lysate prepared from 2.5×10^6^ cells was separated by SDS-PAGE, and then transferred to a PVDF membrane. The membrane was probed with an anti-GFP antibody (Abcam) at a final concentration of 1 µg/ml, followed by goat anti-rabbit HRP-conjugated IgG (Vector Laboratories). The chemiluminescence signals were detected using Immobilon Western Chemiluminescent HRP Substrate (Millipore) and LAS-3000 (FUJI FILM).

### Fluorescent Microscopy and Flow Cytometry

Images were captured using a fluorescence microscope (BX51; Olympus) with a wide bandpass filter set (U-MWIB2; Olympus) and a three charge-coupled device (CCD) camera system (C7780-10; Hamamatsu Photonics) as described previously [19]. Fluorescent intensities of GFP were measured using JSAN cell sorter (Bay bioscience) with 488-nm laser.

## Supporting Information

Table S1 **Primers used in the current study.**
(PDF)
