# Supplementary material for: Gene Targeting in the Red Alga Cyanidioschyzon merolae: Single- and Multi-Copy Insertion Using Authentic and Chimeric Selection Markers
Source: PLoS One. 2013 Sep 5;8(9):e73608. doi: 10.1371/journal.pone.0073608 (PMC3764038; doi:10.1371/journal.pone.0073608)
Supplement: Table S1 — Primers used in the current study. (PDF) [file pone.0073608.s001.pdf]

Supplementary Table S1. Primers used in the current study.

No.	Primers	Sequence 5' to 3'
Primers used in cloning and transformation		
1	URA5.3(-897)F	GAACTGAGGGGCGAACGCA
2	URA5.3(+471)R	CCCTAGCAGCTGACTGTATC
3	D184(773)pqeF	CACCATCACCATCAC GCGTGAGTCAGTTCACTGAC
4	D184(+1884)pqeR	AAGCTCAGCTAATTACAGCTTGCTGACCTTACCC
5	pQE_R	GTGATGGTGATGGTGATGGG
6	pQE_F	TAATTAGCTGAGCTTGGACTCCTG
7	D184(+25)o250R	TATAC GTTCT CGTCG CGTCACCCTCGGGACTTG
8	D184(+28)btF	GCAGGCAAAAAGTGT GAAACCGCTCAGCGACCA
9	O250(-600)pqeF	AAGCTCAGCTAATTACGACGAGAACGTATAAGGAGTG
10	O250(-1)gfpR	GCCCTTGCTCACCATGGTCAACGAACGAAGAAACACA
11	GFP(1)F	ATGGTGAGCAAGGGCGAG
12	GFP(717)R	CTTGTACAGCTCGTCCATGC
13	bt(+1)gfpF	GACGAGCTGTACAAGTAAACTAGCTATTTATCTGGTACAT ATCATTC
14	bt(+200)pqeR	CACCATCACCATCACACACTTTTTGCCTGCACAAGT
15	O250(-600)F	CGACGAGAACGTATAAGGAGTG
16	bt(+200)R	ACACTTTTTGCCTGCACAAGT
17	bt(+200)uraR	TTCGCCCCTCAGTTCACACTTTTTGCCTGCACAAGT
18	D184(+28)uraF	AGTCAGCTGCTAGGGGAAACCGCTCAGCGACCA
19	D184(1270)F	ACACGAATCACACGGTGCTG
20	D184(+1448)R	TTGCCGATAACGCAGAAGAGA
21	URA5.3(1412)R	AGGTCGCTGATGCGGAA
22	URA5.3_(+3)F	GAATACGTTGAATGATTCCTAATGGGCAGAAGCAAG
23	GsURA5.3F	CCGCATCAGCGACCTTGTCGGAACACTCCGCC
24	GsURA5.3R	TCATTCAACGTATTCTTCAAGTCGTTG
Primers used to check for homologous recombination		
25	D184_773F (F1)	CACCATCACCATCACGCGTGAGTCAGTTCACTGAC
26	GFP_717(R1)	CTTGTACAGCTCGTCCATGC
27	URA_+3(F2)	GAATACGTTGAATGATTCCTAATGGGCAGAAGCAAG
28	D184_+1884(R2)	AAGCTCAGCTAATTACAGCTTGCTGACCTTACCC
29	D184_+25(R3)	CGTCACCCTCGGGACTTG
30	D184_+28(F3)	ACGAATGTTCCTAGAGAAACCGCTCAGCGACCA
Primers used in preparation of a probe in southern blot analysis		
31	$URA_{Cm}F$	CCTTTTGCGACAGCAATGG
32	$URA_{Gs}R$	GACCCACTTCATTGGCAATG
Primers used in q-PCR analysis		
33	D184(773)Fa	CACCATCACCATCACGCGTGAGTCAGTTCACTGAC
34	D184(873)Ra	ATACCCGGGCACAGGAAC
35	D184(2659)Fb	TACAAGGAACCAGTTGAGGGT
36	D184(+25)Rb	CGTCACCCTCGGGACTTG
37	GFP(638)Fc	ACGAGAAGCGCGATCACA
38	GFP(717)Rc	CTTGTACAGCTCGTCCATGC

Underlines indicate adaptor sequenses for In-Fusion reaction.
